# Annotations

**Published:** 1893-05-06

**Authors:** 


### Annotations.

Archdeacon Farrar, in his memorial address at St. Margaret's, Westminster, quoted a sentence from one of the late Earl of Derby's speeches which deserves to be remembered among the sayings of great men. In the course of a speech addressed to the students of Glasgow University Lord Derby said: "The man who has only himself to please finds, soon or late, and sooner rather than later, that he has got a very bad master; for where great interests are excluded little matters become great, and petty and imaginary vexations breed and multiply in the unoccupied brain. And there is a moral aspect of these questions. If the matter is to be dealt with *in foro conscientiae*, I think a scrupulous and high-minded man, and especially if he be very wealthy, will always feel that to pass out of the world in the world's debt, to have consumed much and produced nothing, to have sat down, as it were, at the feast, and gone away without paying his reckoning, is not—to put it in the mildest way—a satisfactory transaction." It would be almost impossible to put the appeal of duty to an Englishman in a more effective form than this. To sit down at the feast, and go away without paying the reckoning is the kind of thing which the average Englishman, to say nothing of the high-minded Briton, cannot by any possibility imagine himself doing. He would sink himself into the ground with the weight of his own contempt if he did such a thing. And yet Lord Derby appeared to think that great and rich men might enjoy the whole of life's feast and forget the reckoning, not only during the feast, but at its close. We do not like to appear to diminish the broad justice and majesty of such sentiments by applying them to one particular case, as if there were no other case of equal worth and claim. But we venture to suggest to any man who feels that his reckoning is in arrear, that the care of the sick is always an urgent duty, and that hospitals often have to do much work on little means. In no other way can the rich, who have found life a feast, better pay the reckoning to the poor, who have found it something of a prolonged fast, than by giving very freely to hospitals.

The "Reckoning."

The town of Leeds has been, by its representatives, arguing out the question of "back-to-back" houses before a Select Committee of the House of Commons. What are "back-to-back" houses? the reader may ask who knows nothing of Leeds. They constitute a class of property which illustrates the Yorkshireman's first and most favourite principle of trade. According to a distinguished Yorkshire economist of the operative class, the first principle of trade is to make something out of nothing, or as that particular economic genius expressed it in his native Doric, "To mak summat aht o' nowt." Acting on this principle, some inventive Yorkshireman hit upon the idea of "back-to-back" houses; and his keen-eyed fellow-countrymen at once perceived a brilliant illustration of their favourite principle, and proceeded to make back yards out of nothing; or rather to build houses in such a fashion that back yards became, not merely superfluities, but impossibilities. What then are "back-to-back" houses? If the uninstructed reader will imagine his own villa split into two by a solid wall running through the middle of the house from the basement to the ridge of the roof, and his back door and back windows converted into a front door and front windows, looking in the opposite direction from the *bona fide* front door and front windows, he will have in his mind's eye a picture of two back-to-back houses. In other words, the Yorkshireman builds two houses on the same piece of ground on which a less keen economist ordinarily builds one. Of course, such houses have no back doors, no back windows, and no back yards. Equally, of course, they have no "through draught of air"; and also, of course, when they are of the cottage class their privies, middens, dustbins, and other elegant appanages of Leeds civilisation and health, are often placed immediately facing the front door and the front windows. If anybody were to ask a sanitarian of ordinary intelligence whether such houses could possibly be healthy or decent, the sanitarian would wonder whether his questioner took him for a fool. And yet it is said that four Leeds doctors have been found who have testified before the House of Commons that such houses are quite healthy and fit for human habitation. Now, if the "four philosophers of Leeds" had testified that they were anxious to promote the interests of speculative builders, and were willing to sacrifice the health of the poor in so sacred a cause, we might, at least, have believed them. But such medical testimony as they actually gave is as valueless as we hope "back-to-back" house property will some day be made. The House of Commons seems to have acquiesced in the building of more of such houses, at any rate during a "transitional period." This is a disgraceful piece of folly. Will not the more intelligent inhabitants of Leeds, of which Mr. Pridgin Teale is so distinguished a resident and sanitarian, rouse themselves and deliver their ancient and historic town from knavish builders, who pile up fortunes by ruining the public health, and from their medical fellow-townsmen who make sanitary science a laughing-stock and a by-word among honest, straightforward men?

Leeds and her Back-to-Back Houses.

We publish on another page a letter from "C. M. A.," who calls attention to Dr. Rand's cure for snoring, announced recently in The Hospital, and suggests that some application of it to snorers in general and to nurses in particular would be a "benefit to humanity." "C. M. A." is undoubtedly right; but it would have been more to the point if she had shown exactly how Dr. Rand's cure could be universally applied. When people snore, it is because the current of air, passing through the mouth instead of the nose, into and out of the air passages, is more or less interrupted. The current may be interrupted in a variety of ways, as, for example, by flexion of the neck in the act of bending forward the head, by the falling down of the soft palate, and by other means, which will at once suggest themselves to those who are familiar with the structure of the parts. The problem is, how to compel nose breathing, or so to fix the heads of sleeping mouth breathers as to distend the air passages to their utmost capacity, and to keep them in that position. Obviously it cannot always be done, as a moment's reflection or actual experience will show. For example, a snorer is "blowing like a porpoise," and driving all the other persons in the house frantic with rage. He is waked up, and informed of his misdeeds, and without more ado readjusts his head and throat, and the snoring ceases. But he gradually falls asleep again, with the result that his head involuntarily drops into the old position, and once more the snoring trumpet peals out its maddening strains. The only real cure for snoring is either to compel nose breathing, or to produce such an impression on the mind of the snorer of the enormity of his offence that he will, even in sleep, learn to hear the sound of his own snoring, and will, half involuntarily, readjust his head without waking. Dr. Rand's cure for the apoplectic was perfect in its way, but then it is to be remembered that the apoplectic was unconscious and paralysed, and that the head once fixed in the right position would remain there, other things being equal. But the cure of snorers who snore in health is by no means so simple a business as "C. M. A." seems to imagine.

